# SenseCrypt: A Security Framework for Mobile Crowd Sensing Applications

**DOI:** 10.3390/s20113280

**Published:** 2020-06-09

**Authors:** Nsikak Pius Owoh, Manmeet Mahinderjit Singh

**Affiliations:** School of Computer Sciences, Universiti Sains Malaysia, Penang 11800, Malaysia; manmeet@usm.my

**Keywords:** Internet of Things, mobile crowd sensing, security and privacy, data annotation, signcryption, data compression, message queuing telemetry transport protocol

## Abstract

The proliferation of mobile devices such as smartphones and tablets with embedded sensors and communication features has led to the introduction of a novel sensing paradigm called mobile crowd sensing. Despite its opportunities and advantages over traditional wireless sensor networks, mobile crowd sensing still faces security and privacy issues, among other challenges. Specifically, the security and privacy of sensitive location information of users remain lingering issues, considering the “on” and “off” state of global positioning system sensor in smartphones. To address this problem, this paper proposes “SenseCrypt”, a framework that automatically annotates and signcrypts sensitive location information of mobile crowd sensing users. The framework relies on *K*-means algorithm and a certificateless aggregate signcryption scheme (CLASC). It incorporates spatial coding as the data compression technique and message query telemetry transport as the messaging protocol. Results presented in this paper show that the proposed framework incurs low computational cost and communication overhead. Also, the framework is robust against privileged insider attack, replay and forgery attacks. Confidentiality, integrity and non-repudiation are security services offered by the proposed framework.

## 1. Introduction

The Internet of Things (IoT) is a dynamic and global network infrastructure for linking together the physical and virtual world, using standard and interoperable communication protocols [1]. The IoT uses well-known technologies such as wireless sensor networks (WSNs) and radio frequency identification (RFID). A recent IoT trend is mobile crowd sensing, where carriers (known as a “crowd”) of sensing and computing devices such as smartphones, tablets and wearable devices acquire and share essential data for various applications [2]. Mobile Crowd Sensing (MCS) has revolutionized the IoT to become a vital sensing mechanism. The advancement in mobile technology has been key to the advantages of MCS over traditional sensing technologies (such as WSNs). Firstly, the availability of affordable smartphones with integrated sensors has enabled the development of several landmark applications. Furthermore, the programmability of smartphones supports novel sensing applications such as the sharing of user’s real-time activity with friends on social networks. Secondly, apart from sensing, mobile devices like smartphones have computing and communication features which allow programmers to deploy third-party applications. Thirdly, the availability of application stores by phone vendors allow sensing application developers to ship out novel applications at large-scale. Such large-scale sensing was not possible with previous sensing technologies like wireless sensing networks (WSNs). Fourthly, developers can offload mobile services to backend servers, thereby ensuring additional computing resources that aid advanced features in sensing applications [3]. An example of such sensing applications is feedback and persuasion apps. Sensing applications can be broadly classified into people-centric and environment-centric sensing. People-centric sensing focuses on collecting human-related data at different scales to record users’ activities (e.g., daily routines), health records and analyze behaviours (e.g., gait) [4]. Personal, group and community sensing are categories of this sensing type. Environment-centric sensing, on the other hand, collects data about the environment (e.g., noise and air pollution) [5]. Smartphone sensors such as accelerometers, gyroscopes, magnetometers and GPS recievers aid the development of novel applications across several domains such as transportation [6], healthcare [7], social networks [8], safety [9], and environmental monitoring [10], thereby expanding the applicability of mobile crowd sensing. Despite its benefits, MCS still faces challenges that include security and privacy [11,12,13]; quality and reliability of sensed data [14]; incentivization of participants [15]. Other issues in MCS are energy consumption of mobile sensing devices [16]; and sensor data annotation [17,18,19,20,21].

Security and privacy are pressing issues in MCS, raising concerns about the collection and usage of personal data. In MCS, sensitive information of users such as their identity and location information are vulnerable to privacy attacks [22]. An adversary can intercept MCS traffic and capture the sensitive information of users contained in sensor data. For example, GPS sensor readings can be used by an adversary to obtain personal information about MCS participants, such as their daily routes to work and their home location. With knowledge of the possible vulnerability of their sensitive data, MCS users are mostly reluctant to participate in sensing tasks.

Some frameworks have been proposed to address security and privacy issues in MCS. These frameworks include PRISM, proposed by Das [23]; AnonySense, proposed by Shin [24] and PEPSI, proposed by De Cristofaro and Soriente [25]. These frameworks, however, overlook the security of sensitive location information of participants. To tackle this issue, Liu [26] proposed a security framework called Invisible Hand, which uses economic models to secure location information of MCS users. Nevertheless, the proposed framework, while protecting location information of users does not consider the peculiarity of GPS signals, considering the “on” and “off” state of GPS sensors in mobile devices. The lack of an appropriate automatic annotation mechanism for sensitive location data at the sensing layer of MCS undermines the effective security of such data.

Encryption and digital signature schemes are significant cryptographic primitives used in many applications. Signcryption ensures signing and encryption in one logical step. It incurs low computational cost and communication overhead compared to the sign-then-encrypt technique. Signcryption guarantees confidentiality, integrity, authenticity and non-repudiation. Nevertheless, drawbacks with signcryption include, transfer of a large amount of information and its verification complexity. A remedy to these drawbacks is the aggregate signcryption scheme, which aggregates signcrypted messages, hence reducing the amount of transferred information between communicating entities. This approach minimizes the computational complexity and communication overheads associated with signcryption schemes. Application areas of aggregate signcryption include online banking, online polling, and traffic management. Specifically, certificateless aggregate signcryption (CLASC) have been employed in e-auction [27] and vehicular crowdsensing [28]. However, computational complexity and communication overheads still exist with CLASC schemes due to the implementation approach used.

In this paper, we show that a novel implementation approach involving spatial coding compression and the integration of MQTT can enhance the CLASC scheme proposed by Basudan, Lin [28] in terms of computation and communication overheads. To this end, this paper proposes a framework called “SenseCrypt”, that automatically labels sensor data either as non-sensitive or sensitive data. The framework also signcrypts data labelled as sensitive data. The framework, which is an extension of our previous work in [21], employs *K*-means algorithm for the annotation of sensitive location information and a certificateless aggregate signcryption scheme for data security.

The contributions of this paper are threefold:

1. To propose an annotation model that labels sensor data into non-sensitive (does not contain location readings) or sensitive (contains location readings) clusters.
2. To secure sensitive location data in MCS using an efficient CLASC scheme that incurs low computational cost and communication overheads.
3. To evaluate the performance of the proposed framework against known attacks in mobile crowd sensing.

The rest of this paper is structured as follows: Section 2 presents a review of related works on some security and privacy techniques and frameworks in MCS. In Section 3, we present our proposed SenseCrypt framework that incorporates data annotation and sensitive data signcryption. Furthermore, the dataset used to develop the models in the framework are also discussed in this section. The results from the evaluation of the framework are shown and discussed in Section 4. The section also presents the performance and security analysis of the proposed framework. Section 5 concludes the paper and highlights our future work.

## 2. Literature Review

Anonymity-based approaches and cryptographic methods are commonly used to ensure security and privacy in mobile crowd sensing [29]. Existing security and privacy solutions that employ these techniques are presented in this section.

### 2.1. Anonymity-Based Techniques in MCS

Anonymity-based methods presented in this subsection include *K*-anonymity, cloaking, pseudonymity, and differential privacy.

#### 2.1.1. *K*-Anonymity

*K*-anonymity in MCS is an anonymisation technique that removes unique details in the information of *K* participants by adding similar information from other participants [30]. Consequently, *K*-anonymity is ensured in the generated information if the data for each participant cannot be identified from at least *K*-1 other participants [31]. This privacy approach is implemented in Privacy-Preserving Reputation System (PPRS), which was proposed by Huang [32]. The system comprises of participants, a trusted third party and an application server as entities. The system employs *K*-anonymity on participant’s location and time data by normalising them with similar data of other participants. The trusted third party in the framework ensures the security and privacy of participants’ data and guarantees data trustworthiness. However, *K*-anonymity is still vulnerable to homogeneity attacks. These attacks exploit the monotony of certain features to identify users from the set of *k* participant [4].

#### 2.1.2. Cloaking

Cloaking is a technique that replaces actual data with their corresponding anonymised versions to avoid unique identification of the real data [32]. A common cloaking technique is spatial cloaking. In spatial cloaking approaches, sensitive information is blurred in a cloaked zone, thereby maintaining users’ privacy. Spatial cloaking employs generalisation, transformations, or fake locations to hide the actual location of participants. A spatial cloaking technique is used by Ghinita [33]. The authors segment spaces into a set of regions, then employ a specific probability distribution to select participants in each region to broadcast their precise location. Also, Kazemi and Shahabi [34] employ a peer-to-peer spatial cloaking technique to cloak users’ location when querying MCS servers. Nonetheless, major issues with this technique are that a single point of failure is possible. Secondly, to enforce cloaking, users must continuously forward their locations to the anonymiser, which introduces bottlenecks and delays.

#### 2.1.3. Pseudonymization

Pseudonyms are used to preserve the anonymity of MCS participants by substituting their identities with aliases. In [35], the authors proposed a scheme that uses multiple pseudonyms for individual users and reputation values are sent between various pseudonyms owned by the same user. A trusted server is integrated into the scheme to coordinate the transfer of reputation scores between several pseudonyms. Ma [36] proposed a pseudonym-based anonymous identity authentication mechanism for MCS. It uses pseudonym construction rules to encrypt the real names of MCS users. The security mechanism is a hybridisation of public key infrastructure (PKI) and combined public key (CPK) technology to address the large-scale key management issue. The identity authentication mechanism proposed by the authors consists of an application server, a certificate authority database, and a key management centre (KMC). The key management centre employs elliptic curve cryptography (ECC), which sets an m×h order to the secretive seed key (SSK) of integer vector $\left(r_{ij}\right).$ It then computes a public key vector $\left(r_{ij}G\right)=\left(x_{ij},y_{ij}\right)$ to obtain a public seed key (PSK) matrix. G in the scheme is the base point of the elliptic curve, while x and y are the public key parameters. The security scheme then publishes PSK, while keeping SSK private. However, pseudonyms must be complemented by other security mechanisms to effectively secure location information of participants, which makes it a non-trivial approach [4].

#### 2.1.4. Differential Privacy

A general issue with cloaking techniques is its ineffectiveness when an attacker has prior knowledge of the user’s location distribution [37]. Differential privacy [38] is employed in the location privacy-preserving framework proposed by Wang [39] as a solution to the abovementioned problem. The framework integrates location obfuscation and data adjustment to achieve secure privacy. Real-life traffic monitoring and temperature datasets were used to evaluate the proposed scheme. Results show that the proposed scheme evenly distributes obfuscation and improves the inference accuracy of the obfuscated data. Furthermore, Wang [40] employed differential privacy into task allocation to ensure the security of location privacy irrespective of adversaries’ prior knowledge of the data. With the scheme, participants can obfuscate their reported locations without the aid of any third-party. A summary of some proposed security schemes that adopt the anonymity approach is presented in Table 1.

Anonymity-based techniques are trivial and implementable in MCS. Nevertheless, users’ information can still be linked to their identities, resulting in the de-anonymisation of users [32]. In works proposed by Liu [26] and Zhang [41], the authors showed that malicious entities can infer important information of participants even when participants anonymously sense and process data. With this, even anonymised participants are still vulnerable to location-based inference attacks and tracing attacks [42].

### 2.2. Cryptographic-Based Techniques in MCS

Cryptography is another approach that can be used to achieve security and privacy in MCS by encrypting sensed data at the sender’s side, then transmitting the encrypted data to the application server [30]. Cryptographic techniques ensure that sensitive information of participants remains undisclosed to unauthorised parties. It is a technique that maintains data confidentiality, integrity, authentication and non-repudiation. Cryptographic primitives guarantee security without adding noise to sensor data, thus maintaining its original quality [42]. Some of these primitives are presented below.

#### 2.2.1. Homomorphic Encryption

Several studies have proposed homomorphic encryption as an effective technique in securing sensitive data in MCS. A cloud-enabled privacy-preserving truth discovery (PPTD) framework was proposed by Miao [42]. The PPTD framework ensures effective privacy and high accuracy. The framework employs homomorphic encryption to transmit encrypted sensor readings to the cloud server. After that, users’ encrypted weights are updated by the cloud server without decrypting them, then sends the results to each user. However, the proposed scheme is non-trivial as it incurs significant computation and communication overhead due to the use of fully homomorphic encryption. To improve on their earlier proposed PPTD framework, [43] proposed a novel lightweight privacy-preserving truth discovery framework. The framework employs additive homomorphic encryption to secure sensor data of participants in MCS. Encryption is not directly applied on data, somewhat random numbers are used instead, and the encrypted data is moved to the cloud. A data requester and participating workers are the two components in the proposed framework. A secure system in mobile crowd sensing that utilises both additive homomorphic encryption with garbled circuits was presented by Zheng [44]. Garbled circuits are used to construct the encryption protocol by enabling $S_{0}$ holding $E_{pk1}\left(a\right)$ and $E_{pk1}\left(b\right)$ to get $E_{pk1}\left(a/b\right)$, without disclosing a,b (where *a* and *b* are fractional integers). In this case, ${\mathrm{pk}}_{1}$ is the public key of $S_{1}$. The goal is for $S_{1}$ to generate a garbled circuit for the secure division, while $S_{0}$ evaluates the garbled circuit and finally gets the division result in the encrypted form using $E_{pk1}\left(a\right)$ and $E_{pk1}\left(b\right)$ as inputs. $S_{0}$ and $S_{1}$ are the sensors in the proposed scheme. Homomorphic encryption schemes offer confidentiality, integrity and privacy as security services, but authentication and non-repudiation are not provided. Also, the technique is computational expensive to implement on MCS devices.

#### 2.2.2. Certificateless Aggregate Signcryption

Certificateless public key cryptography (CLPKC) is an intermediate between the traditional public key cryptography (PKC) and ID-based cryptography (ID-PKC) [45]. On the one hand, a certificate authority is required in traditional PKC to generate and manage keys of users, which introduces the certificate management problem. On the other hand, in ID-PKC, the generation of private keys of users based on their identities is done by a trusted key generator. This approach, however, leads to the key escrow problem. CLPKC offers a solution to the mentioned problems. Though CLPKC requires a key generation centre (KGC) for the generation of partial private keys of users, the final private keys are chosen by the users and cannot be accessed by the KGC. The final private keys of users are obtained from the combination of the partial keys generated by the KGC and the secret information selected by the users [27]. Also, the KGC computes the public key of the users using its public parameters with other information, which are secretly kept and published by the user.

Signcryption [46] is a cryptographic primitive that incurs lower computational cost and communication overhead than the sign-then-encrypt technique. In 2008, Barbosa and Farshim [47] proposed the first certificateless signcryption (CLASC) scheme. However, Selvi [48]} proved that Barbosa’s scheme was forgeable. A different CLSC scheme proposed by Liu [49] was shown to be insecure by Weng [50] and Miao [51].

Aggregate signcryption was first conceptualised by Selvi [48], and the authors defined an adequate security model for identity-based aggregate signcryption schemes. They also proposed examples that seem secure in the random oracle model. A security model for certificateless aggregate signcryption schemes (CLASC) was proposed by Eslami and Pakniat [27]. The scheme was proven to be secure in the random oracle model under the gap Bilinear Diffie-Hellman and Computational Diffie-Hellman Intractability assumptions. Basudan [28] proposed another CLASC scheme which enhanced the pairings required by aggregate signature verifications and unsigncryption. The authors used the scheme to develop a privacy-preserving protocol for the improvement of security in data transmission of vehicular crowd sensing. The secure data is used in road surface condition monitoring. The authors showed that their proposed scheme ensures confidentiality, integrity, mutual authentication, privacy and anonymity. However, their scheme still requires implementation enhancement for optimal performance when adopted in generic frameworks. Some proposed cryptographic-based schemes and techniques in MCS and the IoT are summarized in Table 2.

This paper addresses the problems above by presenting a security framework that signcrypts sensitive location information of MCS users using an efficient CLASC scheme. Hence, ensuring data confidentiality, integrity, authentication and non-repudiation.

## 3. Methods

In this section, first, we present the architecture of the proposed framework and discuss the interaction between the different modules. We then divulge the implementation process of the framework.

### 3.1. Architecture of the Proposed Framework

**Figure 1:**
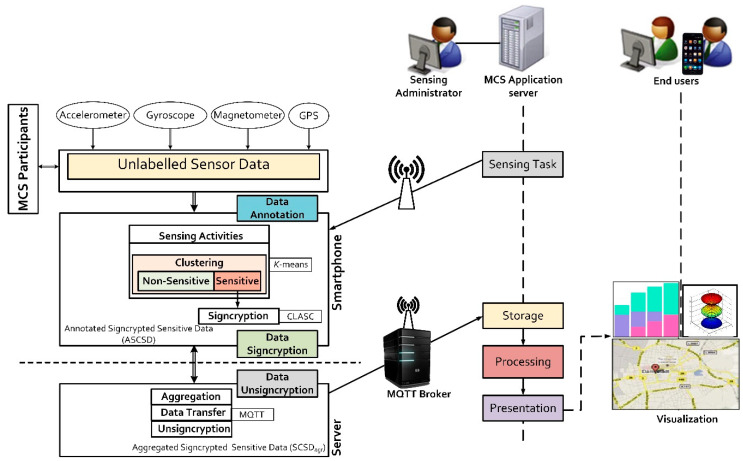
A system diagram of our SenseCrypt framework and its interactions with the standard MCS architecture.

The proposed framework is an enhancement of the typical MCS architecture proposed by Christin [4]. Figure 1 illustrates our system architecture. At the core, our framework consists of two entities: the data annotation and data signcryption modules, which are implemented as a client-server model. The SenseCrypt framework interacts seamlessly with existing MCS stakeholders: sensing administrators, the participants and the end-users:

1. Sensing administrators: they are members of an organization (profit/nonprofit), research groups, individuals who initiate sensing tasks. They design, implement, deploy, manage and maintain MCS applications (using MCS application servers). They set up the application server to acquire, store, and process raw sensor data from participants.
2. Participants: download and install sensing applications on their smartphones and participate in sensing tasks. They collect people-centric or environment-centric data during sensing activities. At a personal scale, these participants may capture data to improve their health conditions or track their sport experiences. Meanwhile, at a community scale, they may upload data to help other users by reporting road and/or traffic conditions. Most times, this information contains sensitive data of participants. Such information requires adequate security against attacks and unauthorized access.
3. End-users: access the data collected by participants based on their needs and preferences. Sensing administrators, participants and other users are all regarded as end-users. End-users visualize processed data by querying MCS application servers which are run by the sensing administrators.

The data annotation module consists of smartphones with integrated accelerometer, gyroscope, magnetometer and GPS sensors as presented in Figure 1. During sensing activities, unlabelled data from these sensors are automatically labelled either as non-sensitive or sensitive data by the framework. This process is achieved using the *K*-means algorithm. All sensor data labelled as sensitive (raw sensor data containing location readings) are compressed then signcrypted using the spatial coding scheme and certificateless aggregate signcryption scheme, respectively. The signcrypted sensitive data is forwarded to the aggregators for aggregation. On the other hand, the MQTT broker handles all published “topics” in the framework and manages communication between publishers and subscribers. Meanwhile, the MCS server is a multi-threaded server system. New threads are used for incoming connections from MCS participants (referred to as mobile clients in later sections of this paper).

**Figure 2:**
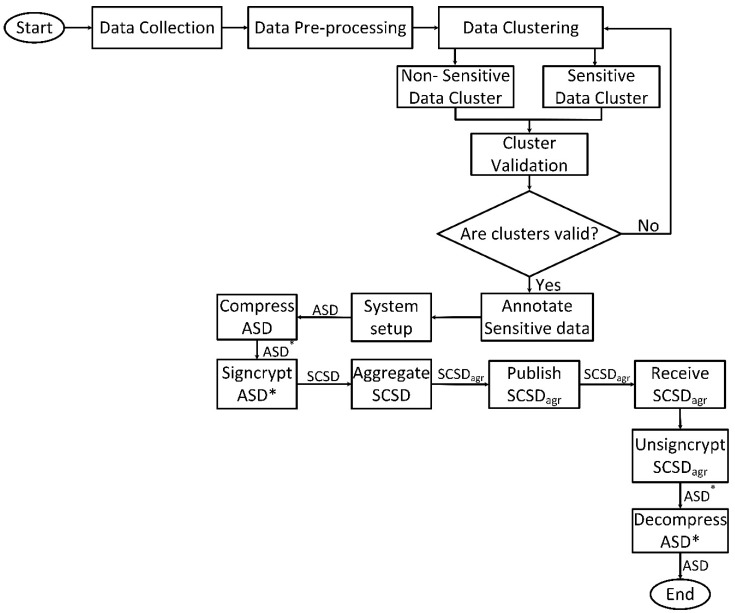
Flowchart of the SenseCrypt framework.

Figure 2 presents the implementation processes of the framework. The process starts with data collection (publicly available dataset) and ends with data decompression. Data signcryption starts after sensitive data have been labelled and validated. It is worthy of note that the data of interest in the framework is contained in the sensitive data cluster denoted as ASD (annotated sensitive data). The ASD is then compressed into ASD*. The compressed annotated sensitive data ASD* is then signcrypted to obtain SCSD (signcrypted sensitive data) and forwarded to the aggregators. After aggregation, SCSD_agr_ is generated and sent to subscribed topics running on the MQTT broker. The MCS application server receives and unsigncrypts SCSD_agr_ to obtain the compressed annotated sensitive data (ASD*). As a final step, the ASD* is decompressed to get the annotated sensitive data ASD.

### 3.2. Sensor Data Annotation

Processes in the sensor data annotation module include data collection (dataset), pre-processing and data clustering.

#### 3.2.1. Dataset

The dataset presented by Freedman [52] was used for the evaluation of the proposed framework. The real-world dataset is a collection of unlabelled motion and location readings of twenty participants acquired over six months. There are 3112 instances and 36 attributes in the dataset. After feature selection and extraction, 11 relevant attributes were obtained including accelerometer (Ax, Ay, Az), gyroscope (Gx, Gy, Gz), magnetometer (Mx, My, Mz) and GPS (latitude, longitude). Table 3 presents the extracted features and their description. The availability/unavailability of GPS data in terms of outdoor and indoor movements of users captured in the dataset makes it appropriate for the framework. Since the first task of the framework is to model the fluctuations of GPS sensor and automatically label any sensor reading containing location data as sensitive, otherwise non-sensitive, dataset presented by Freedman [52] meets this purpose.

Furthermore, data normalization was performed as a pre-processing process since the data range of some features in the dataset is enormous, and such dimensions determine the variance of the distance. The Min-Max normalisation method was used to ensure that all the data values come under the range of (0,1).

#### 3.2.2. *K*-Means Clustering

The *K*-means algorithm is an unsupervised learning algorithm commonly used in tackling clustering problems in sensor networks due to its simple implementation and linear-complexity [53]. It separates data into different groups (referred to as clusters). With *K*-means clustering, cluster centres (C) are stochastically initialized to K from points in a given data to ensure uniqueness of all centroids (i.e., $\forall \;centroids\;C_{i}\;and\;C_{j},\;C_{i}\neq C_{j}$). For the *K*-means to function, three parameters must be provided by the user, which are: number of clusters K, cluster initialisation and the distance metric [54]. The *K*-means algorithm can be formally represented as follows:

Let $D=\left\{d_{1},\ldots ,d_{n}\right\}$ be the data (sensor data), $\mu_{q}=\underset{d\epsilon C_{q}}{\sum }\left(d|n_{q}\right)$ be the centroid of the cluster $C_{q}$ and let K be the cluster number (1≤q≤K). Then the objective function of the *K*-means clustering algorithm is the sum of squared error (SSE) as follows:

(1) $$S_{k}=\overset{K}{\underset{q=1}{\sum }}\underset{d\epsilon C_{q}}{\sum }\;∥d-\mu_{q}{∥}^{2}$$

where $\mu_{q}$ is the mean of the cluster $C_{q}$ containing data points $\left\{d_{1},\ldots ,d_{n}\right\}$ and d is a high dimension set of observations. The aim here is to minimize the objective function for a fixed number of clusters. The *K*-means algorithm used to run the pre-processed dataset, distinctly grouped sensor data into groups. The clustering model from the *K*-means algorithm, which was implemented in Python runs on the smartphone. The model was used to annotate sensor data within the dataset into non-sensitive and sensitive clusters. This process is performed on the client-side (smartphone) before the compression and encryption of location data (data in the sensitive cluster).

### 3.3. Sensitive Data Signcryption

The data signcryption module of the proposed SenseCrypt framework employs the certificateless aggregate signcryption scheme. This subsection presents the CLASC scheme and its implementation in the system model.

#### 3.3.1. Preliminaries of the Certificateless Aggregate Signcryption (CLASC)

This subsection first provides an overview of the bilinear pairing definition, which is adopted in the CLASC scheme for the proposed SenseCrypt framework.

*Bilinear Maps*: Let $G_{1}$ be an additive group of large prime order q, and $G_{2}$ be a multiplicative group of similar large prime order. Then let $G_{1}$ be generated by P. With this, an admissible bilinear pairing $\tilde{e}:G_{1}\times G_{1}\to G_{2}$ is a map that has the following properties:

1. Bilinearity: For all $P,Q\in G_{1}$ and $a,b\in Z_{q}^{*},$ then $\tilde{e}\left(aP,bQ\right)=\tilde{e}{\left(P,Q\right)}^{a}{}^{b}$
2. Computability: An algorithm that computes $\tilde{e}\left(P,Q\right)$ for $P,Q\in G_{1}$ is efficient. $\tilde{e}:G_{1}\times G_{1}\to G_{2}$, which is an admissible bilinear pairing can be run using the modified Weil/Tate pairing over elliptic curves [55].
3. Nondegeneracy: $\tilde{e:}\left(P,Q\right)\neq 1G_{2}$ where the identity element of a group $G_{1}$ is represented with $1_{G_{2}}$.

*Definition of bilinear Parameter Generator*: A bilinear parameter generator *Gen* is a probabilistic algorithm that accepts input k as a security parameter and generates as outputs a 5-tuple $\left(G_{1},G_{2},\tilde{e},P,q\right),$ where $G_{1}.G_{2}$ are two groups with order q, $\tilde{e}$ is a non-degenerated and trivial bilinear map, $P\in G_{1}$ is a generator and q is a *k*-bit prime number.

The components of CLASC are defined based on initial theories proposed by Lu and Xie [56] and Eslami and Pakniat [27]. These components are: a Key Generator Centre (KGC), an aggregating set of $ID_{i}$ of *n* users with identity of ${\left\{ID_{i}\right\}}_{i=1}^{n};$ recipient(s) represented with the identity $ID_{R}$ with an aggregate signcryption generator. Therefore, the following seven probabilistic polynomial time algorithms [28] defines the CLASC scheme of the SenseCrypt framework:

1. *Setup*: An algorithm that accepts k input as a security parameter, outputs *SysParams* as system parameters and a master key s, an associated master public key $Y_{pub}$. The KCG then implements the algorithm and publishes *SysParams* and securely stores the key.
2. *Partial-Private-Key-Extract*: Given the system parameters *SysParams*, s and identity $ID_{i}$ of an identity i. A partial private key $F_{part}$ is generated by the algorithm and forwarded by the KGC to the legitimate user i.
3. *User-Key-Generate*: Each user implements this algorithm and accepts inputs *SysParams* and $ID_{i}$ (user’s identity). The output from this algorithm is a randomly selected secret value $g_{i}$ with an associated public key $X_{i}$. The public key is generated and published by the user.
4. *Signcrypt*: Each user $ID_{i}$ which is a member of the aggregated set of n users ${\left\{ID_{i}\right\}}_{i=1}^{n}$ runs this algorithm. Δ is accepted as the state information together with *SysParams*. All the users must employ similar but distinct state information in the signcryption algorithm. A message $m_{i},$ user’s identity $ID_{i}$ must be used by all users with the associated public key $X_{i}$ and a private key pair $\left(g_{i},F_{part}\right)$, $ID_{R}$ (receiver’s identity) and with the associated public key $X_{R}$. With this, the ciphertext $C_{i}$ is generated.
5. *Aggregate*: The aggregate signcryption generator runs this algorithm and accepts the following inputs: an aggregating set of $ID_{i}$ of n users’ ${\left\{ID_{i}\right\}}_{i=1}^{n}$, Δ (state information), each sender’s identity $ID_{i}$ with the associated public key $X_{i}$ and $C_{i}$ (cipher generated from the message $m_{i}$). Next, the state information Δ, with the associated public key $X_{R}$ and the receiver’s identity $ID_{R}$ are applied to the message to generate a cipher. The output is an aggregated ciphertext C on messages ${\left\{m_{i}\right\}}_{i=1}^{n}.$
6. *Aggregate-verify*: The receiver $ID_{R}$ runs this algorithm by accepting as input an aggregating set of n users ${\left\{ID_{i}\right\}}_{i=1}^{n},$ the sender’s user identity $ID_{i}$ state information Δ, an aggregated ciphertext C and the associated public key $X_{i}.$ The algorithm only returns true if the aggregate signcryption is legitimate, else it returns false.
7. *Aggregate*-Unsigncrypt: This algorithm is run by $ID_{R}$ (the receiver) and accepts as input an aggregated ciphertext C, the receiver’s entire private key $\left(g_{R},F_{part\left(R\right)}\right)$, receiver’s identity $ID_{q}$, the senders’ identities ${\left\{ID_{i}\right\}}_{i=1}^{n}$, public key $X_{R}$ with their corresponding public keys ${\left\{X_{i}\right\}}_{i=1}^{n}$ and the state information Δ. The algorithm then returns a set of n plaintexts ${\left\{m_{i}\right\}}_{i=1}^{n}.$

#### 3.3.2. The CLASC Scheme

This subsection presents the CLASC scheme of the SenseCrypt framework. Table 4 shows the mathematical notations used in the CLASC scheme.

#### 3.3.3. System Model

**Figure 3:**
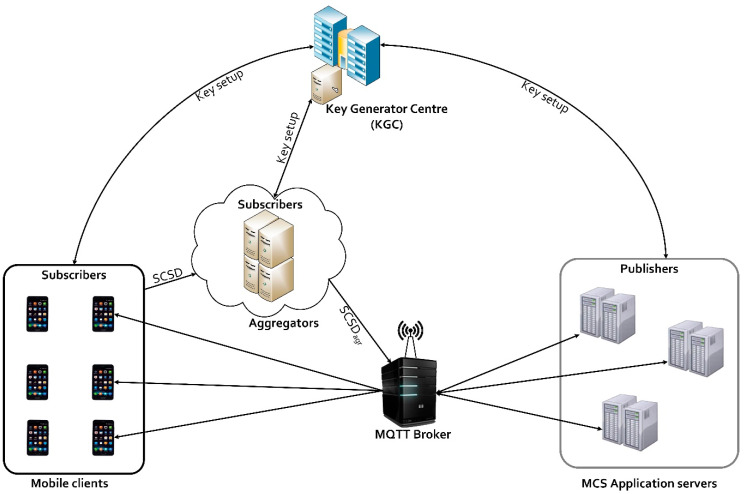
Model implementation of the CLASC scheme.

We present an efficient implementation approach for the CLASC scheme proposed by Basudan [28]. The scheme is designed to ensure the signing and encryption of annotated sensitive data (ASD) in one logical step. The components of the model are shown in Figure 3. The key generator centre (KGC) is a trusted third party entity that only generates a partial private key for mobile clients (MC), aggregators (*AG*) and the MCS application server (AS) but does not have access to their final private keys, hence, cannot access sensor data transmitted between them. Implementing this eliminates the key escrow problem and ensures that sensitive location information of MCS users remains private.

On the other hand, (MC) are users that employ smartphones to collect sensor data which contain their sensitive location information. Data compression is performed on annotated sensitive data (ASD) before signing and encryption. After signing and encryption of the ASD, the signed ciphertext is forwarded to the aggregator (*AG*) using their respective partial private keys. The (*AG*) aggregates signed ciphertexts then forwards the aggregated ciphertexts to the MQTT broker as subscribed “topics”. The MCS application server (AS) receives the aggregated ciphertexts from the MQTT broker via its published topics.

The (AS) then verifies, unsigncrypts the aggregated ciphertexts using his associated private key. The CLASC scheme implemented in the SenseCrypt framework comprises of the following steps: (i) system setup; (ii) data compression; (iii) annotated sensitive data signcryption; (iv) signcrypted sensitive data aggregation; (v) efficient data transfer; (vi) receive signcrypted sensitive data.

##### System Setup

First, the key generator centre (KGC) registers both the mobile client (*MC*), the aggregator (*AG*) and the MCS application server (*AS*). Then generates partial private keys $F_{part}$;$F_{part\left(R\right)}$ and public keys key $X_{i}$; $X_{R}$ for $MC_{i},\;AG_{j}$ and $AS_{R}$, respectively. Next, the KGC generates the bilinear parameters $\left(G_{1},G_{2},\tilde{e},P,q\right),$ given the security parameter k, and performs this by executing *Gen(k)*. Then, the KGC chooses at random $s\in Z_{q}^{*}$ as its master secret key and computes the master public key $Y_{pub}=sY$. Furthermore, four secure hash functions are selected by the CU: $H_{1}:{\left\{0,1\right\}}^{*}\to Z_{q}^{*},H_{2}:{\left\{0,1\right\}}^{*}\to {\left\{0,1\right\}}^{\ell }$ where ℓ is the bit-length of plaintexts, $H_{3}:{\left\{0,1\right\}}^{*}\to G_{1}$ and $H_{4}:Z_{q}^{*}\to G_{1}$ [28]. At this point, the system parameters *SysParams*, $\left(G_{1},G_{2},\tilde{e},P,q,Y_{pub},H_{1},H_{2},H_{3},H_{4}\right)$ are available to the registered users $MC_{i}$, $AG_{j}\;and$ $AS_{R}$. The entire setup process for $MC_{i}$ $AG_{j}$ and $AS_{R}$ is shown as follows:

1. A mobile client $MC_{i}$ can arbitrarily select $g_{i}\in Z_{q}^{*}$ as its secret value, then computes its partial public key $MC_{i\left(a\right)}=g_{i}P.$
2. To preserve privacy, $MC_{i}$ can pseudonymize its identity by randomly selecting $Q_{i}.$
3. $MC_{i}$ forwards its identity and partial public key $\left(MC_{i},MC_{i\left(a\right)}\right)$ to the KGC for registration.
4. The KGC arbitrarily chooses $g_{i}\in Z_{q}^{*}$ and computes a different partial public key for *MC:* $MC_{i\left(b\right)}=g_{i}P.$
5. KGC calculates the partial private key $F_{part}{}_{i}=g_{i}+s*Q_{i},$ where $Q_{i}=H_{1}\left(MC_{i}\right),$ this registers $MC_{i}$ with the partial public key $MC_{i\left(a\right)}$.
6. $F_{part}{}_{i}$ is transmitted securely to $MC_{i}$. In the public key database, the entire public key ($MC_{i\left(a\right)},\;MC_{i\left(b\right)}$) is stored by the KGC.
7. $MC_{i}$ gets the partial private key $F_{part}{}_{i}$ and adds it with its secret value $g_{i}$ to generate its entire private key $\left(g_{i},F_{part}{}_{i}\right).$
8. $MC_{i}$ checks the correctness of the partial private key $g_{i}P=MC_{i\left(b\right)}+Y_{pub}H_{1}\left(MC_{i}\right).$

##### Data Compression

Annotated sensitive data (ASD) to be signcrypted are first compressed by the sender using the spatial coding scheme [57]. Compressing the ASD minimizes the number of messages to be signcrypted. After annotation of sensitive location data, the mobile client (*MC*) compresses the ASD in such a way that loss of data precision is minimized. Therefore, computation overhead which is mostly experienced with signcryption of sensor data from sensing devices, is reduced. Using spatial compression, a ratio γ,0≤γ<1 is defined as the sensor data reduction in size, which is relative to the initial uncompressed sensitive data from each mobile client. Spatial coding [57] is an efficient and effective compression technique on continuous reading, such as those acquired by smartphones. It also defines accurately the alphabets of sensor readings, which minimizes data loss during compression. These features of spatial coding justify why it has been adopted for the compression of ASD in the framework. Mathematically, spatial coding can be represented as follows:

(2) $$\gamma =1-\frac{\mathrm{compressed}\;\mathrm{size}}{\mathrm{uncompressed}\;\mathrm{size}}$$

Each mobile client (*MC*) compresses ASD based on their spatial correlation. The spatial compression involves two steps: the client compression (executed on the smartphone by the participant), and the decompression (at the MCS application server).

##### Annotated Sensitive Data (ASD) Signcryption

This process is carried out by the mobile client $MC_{i}$ with the pseudonym $Q_{i}$. The framework identifies annotated sensitive data, compressed using spatial coding as (ASD*). The certificateless signcryption algorithm is then applied on the ASD* to obtain the signcrypted sensitive data (*SCSD_i_*) using the following steps:

1. $MC_{i}$ randomly chooses $r\in Z_{q}^{*}$ and generates $T_{i}=rP,$
2. Computes $Z_{b}=rPW_{rb}$,
3. Computes $Z_{a}=r\left(PW_{ra}+Y_{pub}Q_{i}\right),$
4. Computes $h_{a}=H_{2}\left(ID_{R}\left|\left|Pk_{ra}\right|\right|PW_{rb}\left|\left|\Gamma \left|\left|T_{i}\right|\right|Z_{b}\right|\right|Z_{a}\right),$
5. Computes $W_{i}=h_{a}⊕ASD^{*}{}_{i}$ and computes $h_{b}=H_{3}(ID_{R}\left|\left|K_{ra}\right|\right|K_{rb}\left|\Gamma \left|\left|T_{i}\right|\right|W_{i}\right|Q_{i}.$
  $\left|\left|MC_{i\left(b\right)}\right|\right|MC_{i\left(a\right)}$
6. Computes $h_{c}=H_{4}\left(\Gamma \right)$,
7. Computes $\beta_{i}=F_{part}{}_{i}h_{c}+rh_{b}+g_{i}h_{c}$.

The ciphertext $C_{i}=\left(T_{i},W_{i},\beta_{i}\right)$ is appended to sensor data in the form of a signcrypted sensitive Data, which is: $SCSD_{i}=\left(Q_{i},Signcrypt\left(SensitiveData_{i}\right)\right).$ The ciphertext $C_{i}=Signcrypt\left(SensitiveData\right)$ ⇒ $\left(T_{i},W_{i},\beta_{i}\right)$ is forwarded to the aggregate server (AG).

##### Signcrypted Sensitive Data (SCSD) Aggregation

On successful signcryption of annotated sensitive data, the $MC_{i}$ forwards the ciphertext $SCSD_{i}$ to the aggregators (also called aggregate servers). The aggregate servers are distributed systems with high computational capabilities. These servers aggregate all the ciphertexts from multiple mobile clients. The property provided by the proposed framework allows for numerous aggregations of ${\left\{SCSD_{i}\right\}}_{i}^{n},$ which further reduces the computational cost [28]. The distributed aggregate servers perform ${\left\{SCSD_{i}\right\}}_{i}^{n}$ aggregation and ${\left\{SCSD_{i}\right\}}_{i}^{n}$ batch verification operations each time an $SCSD_{i}$ is received as shown below:

*A.*  *SCSD Aggregation*

Aggregate SCSD is employed to aggregate several ${\left\{SCSD_{i}\right\}}_{i}^{n}$ into a single SCSD, that is, ${\left\{SCSD_{i}\right\}}_{i=1}^{n}$. For sensitive data $SensitiveData_{i}$, given ${\left\{SCSD_{i}\right\}}_{i}^{n}$, $SCSD_{i}=\left(Q_{i},\;Signcrypt\left(Data_{i}\right)\right)$ by mobile clients $MC_{1},\ldots ,MC_{n},$ it is possible to get $SCSD_{agr}(Q_{i},\ldots Q_{n},Signcrypt{\left(SensitiveData_{i}\right)}_{1}^{i}\ldots Signcrypt\left(Data{)}_{i}^{n}\right).$ An aggregate signcryption generator returns the algorithm below:

1. The algorithm collects single ciphertext $C_{i}={\left(T_{i},W_{i},\beta_{i}\right)}_{i=1}^{n}$ generated by ${\left\{MC_{i}\right\}}_{i}^{n}$ with the pseudonym ${\left(Q_{i}\right)}_{i=1}^{n}$ to a receiver $ID_{R}$ with similar state information Δ.
2. Aggregates several signatures by computing $sign_{agr}\overset{n}{\underset{i=1}{\sum }}\beta_{i}.$
3. Outputs aggregated ciphertexts $SCSD_{agr}=(\left(Q_{i}{)}_{i=1}^{n},T_{1}\ldots T_{n},W_{1}\ldots W_{n},Sig_{agr}\right).$

*B.*  *SCSD Batch Verification*

In this step, all the ciphertexts from ${\left\{MC_{i}\right\}}_{i}^{n}$ users are verified concurrently using the batch verification algorithm. Based on the signature aggregation $sig_{agr},$ the sensor datasets ${\left\{SCSD_{i}\right\}}_{i=1}^{n}$ and the associated public keys ${\left(MC_{i\left(b\right)},MC_{i\left(a\right)}\right)}_{j=1}^{n}$ for all the $MC_{i}$ and the receiver’s identity $ID_{R}$ and its corresponding public key $\left(Xk_{ra},XW_{rb}\right)$ using similar state information Δ. The batch verification algorithm verifies the signature through the following process:

1. $h_{b}=H_{3}(ID_{R}\left|\left|Y_{ra}\right|\right|Y_{rb}\left|\left|\Delta \right|\right|T_{i}\left|\left|Q_{i}\right|\right|MC_{i\left(b\right)}||MC_{i\left(a\right)}),$ for i=1,…,n
2. $h_{c}=H_{4}\left(\Delta \right),$

The signature aggregation $Sig_{agr}$ is accepted if:

$$\tilde{e}\left(sig_{agr},P\right)=\tilde{e}\left(\overset{n}{\underset{i=1}{\sum }}\left(MC_{i\left(b\right)}+Y_{pub}Q_{i},h_{c}\right)\right)\;=\tilde{e}\left(\overset{n}{\underset{i=1}{\sum }}T_{i},h_{b}\right)\tilde{e}\left(\overset{n}{\underset{i=1}{\sum }}MC_{i\left(a\right)},h_{c}\right)$$

If the batch verification process is true, then the aggregator accepts the *SCSDs*. In this case, the $SCSD_{agr}$ will be sent to the unsigncryption step. The $SCSD_{agr}$ is forwarded to the MQTT broker as subscribed topics that are published by the ($AS_{R}$). The efficient transfer process of the $SCSD_{agr}$ is discussed next.

Since the MCS server does not offer acknowledgement, and participants do no retransmission of sensor data, communication overhead is reduced to its minimum. It so happens because data is only sent once when using level 0 QoS, which is adopted in the proposed framework. MQTT uses a password and a username to secure the connection between devices, which makes it less robust to attacks. More so, all MQTT communication is transmitted in plaintext. As stated in SHODAN [58], a lot of IoT devices that use MQTT transfer data to each other without employing an encryption protocol. Hence, data are unprotected during transfer. On the other hand, to employ an encryption protocol with MQTT, the encryption protocol requires independent implementation under MQTT [59]. As such, the proposed framework transmits only signcrypted data from participants to the MCS servers, hence, ensuring the security of sensitive data.

##### Efficient Data Transfer Using MQTT Protocol

**Figure 4:**
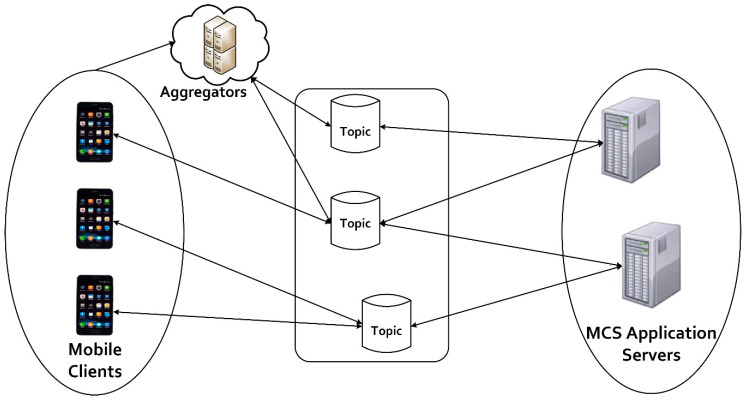
Efficient data transfer using the message queuing telemetry transport protocol.

In the SenseCrypt framework, the transfer of the aggregated sensitive data $\left(SCSD_{agr}\right)$ from aggregators to the MCS application server ($AS_{R}$) is implemented using the MQTT protocol. MQTT, which is based on the publish/subscribe model, incurs less communication overhead and ensures scalability [60]. These properties of the MQTT protocol are leveraged in the SenseCrypt framework. The framework runs a topic-based system, where messages (sensing tasks) are published to topics by the MCS application server. Mobile clients and aggregators subscribe to these topics, as shown in Figure 4. However, in the framework only aggregated signcrypted sensitive data $SCSD_{agr}$ are forwarded to the MQTT broker by the aggregators. The mobile clients subscribe to sensing tasks but forward ciphertexts ${\left\{SCSD_{i}\right\}}_{i}^{n}$ to the aggregator for aggregation and verification. The MQTT broker handles the addition and removal of aggregators and mobile clients (subscribers) from the system and well as performing filtering of forwarded messages ($SCSD_{agr}$).

Since the MCS server does not offer acknowledgement, and aggregators do not retransmit $SCSD_{agr}$, communication overhead is reduced to its minimum. It so happens because data is only sent once when using level 0 QoS (Quality of Service), which is used in the proposed framework. MQTT uses a password and a username to secure the connection between devices, which makes it less robust to attacks. More so, all MQTT communication is transmitted in plaintext. As stated in SHODAN [58], a lot of IoT devices that use MQTT transfer data to each other without employing an encryption protocol. Hence, data are unprotected during transfer. On the other hand, to employ an encryption protocol with MQTT, the encryption protocol requires independent implementation under MQTT [59]. As such, in the proposed framework, aggregators forward signcrypted data $SCSD_{agr}$ from mobile clients to the broker, hence, ensuring the security of sensitive location data of users.

##### Receive Signcrypted Sensitive Data (SCSD)

When the MCS application server receives a message $SCSD_{agr}$, from aggregators via the MQTT broker, it first runs the aggregate-verify algorithm. If the algorithm outputs true, then it implements the next step, which is the aggregate-unsigncrypt. If false, then the $SCSD_{agr}$, is discarded. This process ensures that MCS application servers process only valid signcrypted sensor data from mobile clients that have been aggregated by the aggregators. The $SCSD_{i}$ (Signcrypted Sensitive Data) is decrypted using the following steps:

1. ${\bar{Z}}_{b}=s_{r}T_{i},{\bar{Z}}_{a}=F_{part}T_{i}$
2. ${\bar{h}}_{a}=H_{2}(AS_{R}\left|\left|Pk_{ra}\right|\right|PW_{rb}\left|\left|\Gamma \right|\right|T_{i}{\bar{Z}}_{b}||{\bar{Z}}_{a}).$
3. $SensitiveData_{i}^{\prime }=W_{i}⊕{\bar{h}}_{a}.$

On completion of the decryption process, decompression is initiated. The decompression is carried out by the MCS server. Notably, the MCS server decompresses compressed sensitive data after the unsigncrypt process. Recall that a mobile client $\left(MC_{i}\right)$ compressed annotated data into an array *D* which was a k×k matrix $M=\left(a_{i,j}\right)k\times k,$ such that, each element $a_{i,j}$ stores the value i,j, as discussed earlier in the compression phase. Now to decompress data, the MCS server recovers the associated array D to a two-dimensional matrix which contains $\hat{M}=\left(b_{i,j}\right)k\times k$ Next, the inverse 2D-DCT technique is employed to transform $\hat{M}$ to a new matrix ${\hat{M}}^{\prime }=\left(a_{i,j}\right)k\times k$. The approach employed in the proposed SenseCrypt framework minimizes remarkably the number of transmitted data from mobile clients to the MCS application server. Realizing computational reduction is because sensitive sensor data with high data correlations have been compressed. After data decompression, the actual ASD can be obtained from the ASD*.

## 4. Results and Discussion

This section presents the results from the automatic annotation and signcryption of sensitive location information of MCS users by the proposed SenseCrypt framework.

### 4.1. Performance Evaluation of the Clustering Model

**Figure 5:**
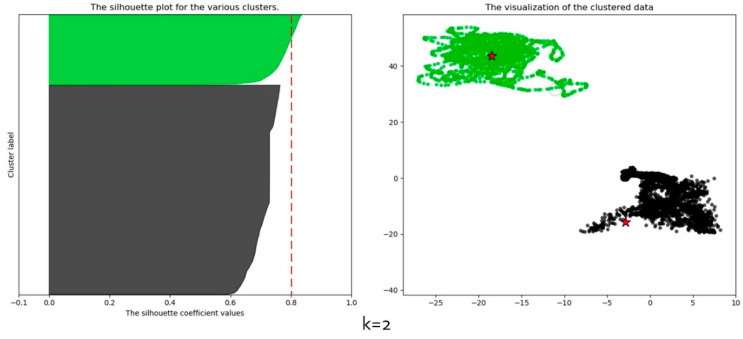
Converged clusters.

For accurate clustering analysis, measuring the distance of objects in the dataset is an important task. There are several types of proximity measures that best fit different types of data. Nevertheless, Euclidean distance and Manhattan distance are mostly used for high dimensional data. In this paper, the *K*-means algorithm was implemented using the Euclidean distance metric. It is a significant metric for identifying the similarity and dissimilarity of generated clusters. It does this by calculating the root of squares between a pair of objects in a dataset. We performed the clustering analysis on 2 to 10 clusters and calculated the average silhouette coefficient against the clustering members. As presented in Table 5, the average silhouette is largest at a value of 0.81, that is when *K* = 2, hence the choice of *K* in the proposed framework. Furthermore, all the clusters are above the average, indicating that data from motion and location sensors in smartphones can be grouped into non-sensitive and sensitive groups. However, the average silhouette decreases slightly as the cluster value increases. Figure 5 shows the clustered data objects from the dataset. As can be seen from the silhouette plot, the non-sensitive data cluster (black colour) contains more data objects than the sensitive data cluster (green colour) based on the thickness of the plots. The clustering results justify the fact that some MCS sensing activities can be performed using only motion sensors since most users prefer to turn off their GPS sensor to preserve battery and/or their privacy. Thus affecting the availability of real-time location data.

The computational complexity of the *K*-means algorithm used in the framework for each iteration is O(i∗n), where n is the number of features in the dataset and i is the value signifying the amount of information from the preceding iterations, which is constant. The number of iterations is 50 and the scale is from 2 to 10. Meanwhile, i was set to 8 and n (number of features) is higher than i (information), which means that the complexity is O(n).

### 4.2. Performance Evaluation of the CLASC Scheme

In this subsection, we evaluate the performance of the CLASC scheme employed in the proposed SenseCrypt framework. We compare our CLASC scheme with schemes proposed by Eslami and Pakniat [27] and Basudan, Lin [28] using computation cost and communication overhead as evaluation metrics. The following parameters are used to measure the scheme’s efficiency:

$T_{p}$: computation time of one pairing operation.
$T_{m}$: computation time of a scalar multiplication point in $G_{1}$.
$T_{e}$: computation time of one exponentiation in $G_{2}$.

**Figure 6:**
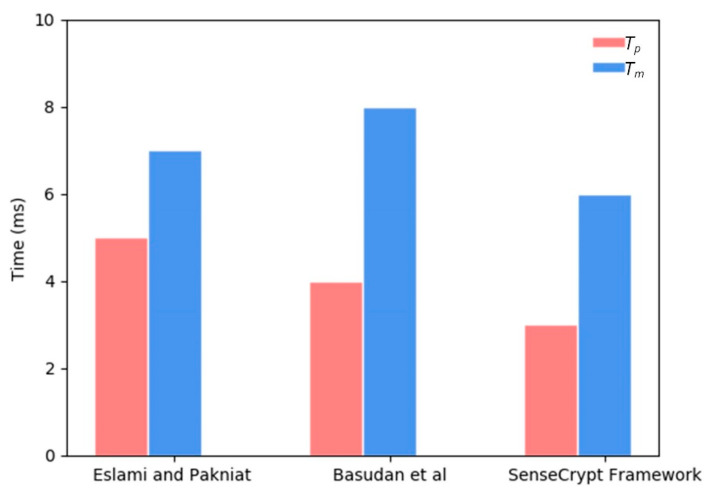
Efficiency evaluation comparison with other CLASC schemes.

The CLASC scheme in the proposed framework allows each $MC_{i}$ to signcrypt sensitive data at any given time. However, mobile clients cannot aggregate signcrypted data, unlike the aggregator that can aggregate multiple signcrypted sensitive data. The effective compression technique adopted in the framework further reduces the size of the sensitive data to be signcrypted. The signcryption algorithm required four multiplication operations in $G_{1}$ for the successful signing and encryption in the framework. A single pairing operation was required for the unsigncrypt process by the MCS application server $\left(AS_{R}\right)$. Table 6 compares pairing time, scalar multiplication time and exponentiation time of the signcryption and unsigncryption processes of our scheme with schemes proposed by Eslami and Pakniat [27] and Basudan, Lin [28]. The comparison can also be visualized in Figure 6. Aggregate verification of the signature and unsigncrypt processes required two scalar multiplication operations. On the other hand, the receiver of the aggregated ciphertext $SCSD_{agr}$ verifies the aggregated signatures in a single step and can verify multiple signatures published in different topics. The number of aggregate signatures can scale based on the scalability property on the MQTT protocol.

The communication overhead of the proposed scheme is derived from the length of the aggregated ciphertext $SCSD_{agr}$ which is the compressed annotated sensitive data $ASD^{*}.$ The MQTT broker in the framework forwards the aggregated ciphertext to the MCS server. Since two parts of each ciphertext $C_{i}$ is required for decryption by the MCS server $\left(AS_{R}\right)$; the communication overhead of the CLASC scheme is a non-constant value. To this end, there exist n+1 elements in $G_{1}$ for the security of the aggregated data $SCSD_{agr}$. Table 7 compares the computational cost and the communication overhead of the schemes discussed above. 

**Figure 7:**
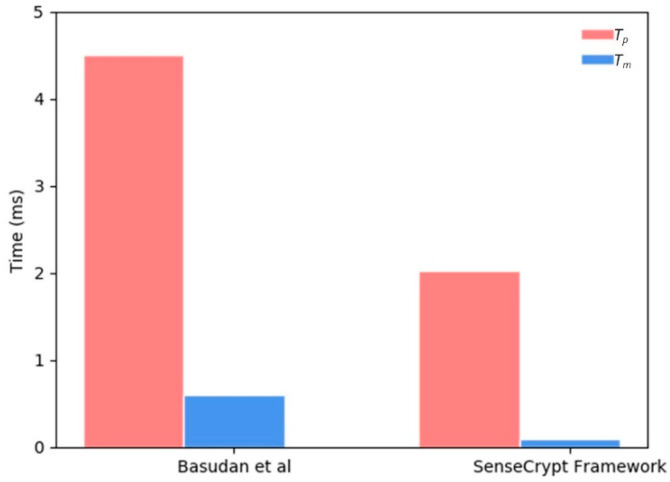
Evaluation of the running time of cryptographic operations.

Similar to the evaluation method used by Basudan [28], we employed an MNT curve [61] and the Weil/Tate pairing $\tilde{e}:G_{1}\times G_{1}\to G_{2}$ curve, where q=160-bit and the degree of curve is 6. To obtain the running time of the cryptographic operations, the scheme was implemented on an Intel Core i7 (TM), 2.90 GHz dual-core machine (simulating operations of the *AS_R_*). Results from the cryptographic operations are presented in Table 8. The running time of our proposed scheme is compared with that of Basudan [28] in Figure 7.

### 4.3. Security Analysis

In this subsection, we analyze the security of the proposed SenseCrypt framework against some known attacks in mobile crowd sensing.

#### 4.3.1. Resilience to Privileged Insider Attack

In the proposed framework, the MCS client $MC_{i}$ forwards his/her signcrypted sensitive data $SCSD_{i}$ to the aggregator. The $SCSD_{i}$ is the ciphertext $C_{i}=\left(T_{i},W_{i}\beta_{i}\right)$, pseudonymized as $SCSD_{i}=\left(Q_{i},Signcrypt\left(SensitiveData\right)\right).$ The pseudonym $Q_{i}$ preserves the identity of $MC_{i}$ from disclosure to the aggregator, MQTT broker and the MCS application server $\left(AS_{R}\right).$ The $Q_{i}$ of $MC_{i}$ derived from a one-way hash function $H_{2}\left(MC_{i}\right)$ protects the identity of $MC_{i}$ from insider attack since the aggregator or $\left(AS_{R}\right)$ does not know the secret value $g_{i}$ chosen by $MC_{i}$.

#### 4.3.2. Resilience to Replay Attack

Timestamps are used to avoid replay attacks in the SenseCrypt framework. Specifically, a timestamp mechanism is employed to ensure the freshness of each published message in the framework. An adversary A, cannot replay the sent $SCSD_{agr}$ to the MCS application server $AS_{R},$ since an ephemeral session key is used for the transfer of $SCSD_{agr}$. Additionally, the authentication message between the $MC_{i}$, aggregator and $AS_{R}$ are protected. Hence, replay attacks cannot succeed in the proposed framework.

#### 4.3.3. Resilience to Forgery Attacks

The first scenario deals with forgery attacks on mobile client $MC_{i}$. In this case, an adversary A may eavesdrop or intercept the message transmitted from $MC_{i}$ in the framework. Then if A sends a forged message to the KGC (Key Generator Centre), the KGC extracts the value of [k] with the secret $rPW_{rb}$ then computes the hash $\left[h_{a}\right].$ The KGC then verifies the legitimacy of the user by checking whether $h_{a}=\left[r\left(PW_{ra}+Y_{pub}Q_{i}\right)\right].$ However, without the knowledge of the correct secret, A cannot compute the valid value [r]. Hence, the framework is secure against $MC_{i}$ forgery attack.

The second scenario is the KGC forgery attack. The message sent from the KGC to the mobile client $MC_{i}$ and MCS application server $AS_{R}$ is protected by the hash mechanism (SHA-256), using the computed key [$F_{part}=g_{i}+s*Q_{i}$]. The adversary A cannot forge the message [$m_{i}$] without knowing [$F_{part}$]. Additionally, without knowing the partial private key, an adversary A cannot forge a valid value [$\left(F_{part}=g_{i}\right)$], which is verifiable by either the mobile client $MC_{i}$ or MCS application server $AS_{R}$. Hence the proposed framework is secure against KGC forgery attack.

#### 4.3.4. User Anonymity and Unlinkability

Users’ identity and location are two major privacy issues of concern for MCS participants. The participant’s real identity is vital in obtaining his/her behaviour. Hence, the participant’s identity and related information must be protected from unauthorized parties. In the proposed framework, the identity of the mobile client $MC_{i}$ is never published over the network, the pseudonym $Q_{i}$ is used instead. This technique makes it impossible for an adversary to reveal the identity of participants from intercepted messages. Since $\left[Q_{i}\right]$ is unlinkable, outsiders or even other participants lack the knowledge of who is communicating with the aggregator or MCS application server at any given time. Hence, the proposed framework prevents identity disclosure and preserves participants’ privacy. Correspondingly, signcrypting the location data of participants $SCSD_{i}$, ensures that only the MCS server $AS_{R}$ with the corresponding private key can unsigncrypt and obtain the plaintext information (sensitive location data) of participants.

#### 4.3.5. Confidentiality and Integrity of Sensitive Location Data

Signcryption of sensitive location data by the mobile client $MC_{i}$ generates a ciphertext $C_{i}=\left(T_{i},W_{i},\beta_{i}\right).$ In this case, $T_{i},W_{i}$ satisfy the encryption properties of the CLASC scheme and $\beta_{i}$ performs signing, all in one step. $AS_{R}$ is the only entity that can unsigncrypt $SCSD_{i}$ (signcrypted sensitive data) through the computation of $T_{i},W_{i},\beta_{i}.$ With this in place, confidentiality is achieved even if an active man-in-the-middle attacker eavesdrops on transmitted sensor data. $SCSD_{i}$ remains undisclosed and cannot be modified, hence ensuring the integrity of sensitive data.

## 5. Conclusions

In this paper, we propose a framework that annotates sensor data and signcrypts sensitive location data of mobile crowd sensing participants. The annotation module of the framework employs the *K*-means algorithm for the labelling of data from multiple smartphone sensors (accelerometer, gyroscope, magnetometer and GPS) into non-sensitive and sensitive clusters. The data signcryption module leverages the signing and encryption properties of the certificateless aggregation signcryption scheme (CLASC) to secure sensitive location data of MCS participants. The paper also puts forward a novel implementation technique that uses efficient data compression technique and MQTT protocol to minimize the computational cost and communication overhead associated with CLASC schemes. Results show that the CLASC scheme implemented in the proposed framework is efficient and robust against attacks such as privilege insider attack, forgery and replay attacks while ensuring confidentiality, integrity and privacy. Presently, the framework only handles location data as sensitive data of interest. As future work, the framework can be extended to incorporate more sensors and annotate other sensitive data in mobile crowd sensing.

## Figures and Tables

**Table 1:** A summary of anonymity-based schemes in MCS.

Anonymity-Based Approaches		
Authors/[Reference]	Techniques	Remarks
[31,32]	*K*-anonymity	Vulnerable to homogeneity attacks, which exploits the monotony of some features to identify users from the set of *K* participants.
[33,34]	Cloaking	An attacker may know users’ location a priori, hence revealing his location.
[35,36]	Pseudonymization	Users’ identities can still be linked from inferred information.
[38,39]	Differential Privacy	Noise added to sensor data reduces data quality.

**Table 2:** A Summary of Cryptographic-based Security Schemes in MCS.

Cryptographic -Based Approaches		
Authors/[Reference]	Techniques	Remarks
[42,43,44]	Homomorphic encryption	Non-trivial (i.e., incurs a high computational and communicational cost). Non-repudiation is not offered.
[27,28]	Certificateless Aggregate Signcryption (CLASC)	Requires enhancement for optimal performance when implemented in a generic framework.

**Table 3:** Extracted Features for Automatic Annotation.

S/N	Features	Description
1.	Ax	Accelerometer X-axis $\left(m/s^{2}\right)$
2.	Ay	Accelerometer Y-axis $\left(m/s^{2}\right)$
3.	Az	Accelerometer Z-axis $\left(m/s^{2}\right)$
4.	Gx	Gyroscope X-axis $\left(m/s^{2}\right)$
5.	Gy	Gyroscope Y-axis $\left(m/s^{2}\right)$
6.	Gz	Gyroscope Z-axis $\left(m/s^{2}\right)$
7.	Mx	Magnetometer X-axis (T)
8.	My	Magnetometer Y-axis (T)
9.	Mz	Magnetometer Z-axis (T)
10.	Lat	Location (Latitude)
11.	Long	Location (Longitude)

**Table 4:** Mathematical Notations I.

Symbols	Description
$G_{1}$	Additive Group
${\left\{m_{i}\right\}}_{i}^{n}$	Aggregated Ciphertext *C* on Messages
$SCSD_{agr}$	Aggregated Signcrypted Sensitive Data
ASD	Annotated Sensitive Data
ℓ	Bit-length of plaintext
$C_{i}$	Ciphertext
$ASD^{*}$	Compressed ASD
⊕	Exclusive OR
P	Group Generator
H	Hash function
${\bar{h}}_{a}$	Hashed message
s	Master private key
$Y_{pub}$	Master public key
$AS_{R}$	MCS Application Server
$m_{i}$	Message
$MC_{i}$	Mobile Client user
$G_{2}$	Multiplicative Group
$\tilde{e}$	Non-degenerated Bilinear map
q	Prime order
r	Random number
$ID_{R}$	Receiver
$ID_{q}$	Receiver’s identity
$\left(F_{part\left(R\right)}\right)$	Receiver’s Partial private key
$\left(g_{R},F_{part\left(R\right)}\right)$	Receiver’s private key
$X_{R}$	Receiver’s Public key
$g_{i}$	Secret value
k	Security parameter
$ID_{i}$	Sender
$F_{part}$	Sender’s Partial private key
$\left(g_{i},F_{part}\right)$	Sender’s private key
$X_{i}$	Sender’s Public keys
$SCSD_{i}$	Signcrypted Sensitive Data
$\left(T_{i},W_{i},\beta_{i}\right)$	Signcryption parameters in the ciphertext
Δ	State information
$Q_{i}$	User’s Pseudonym
$\tilde{e}:G_{1}\times G_{1}\to G_{2}$	Bilinear map
${\left\{ID_{i}\right\}}_{i}^{n}$	Users’ identity

**Table 5:** Data points in generated clusters.

Value of *K*	Silhouette Analysis Score
2	0.81468
3	0.72697
4	0.74805
5	0.66491
6	0.62944
7	0.59680
8	0.59756
9	0.53191
10	0.53458

**Table 6:** Comparison of cryptographic operations with other CLASC schemes.

**Signcrypt**			
**Schemes**	$T_{p}$	$T_{m}$	$T_{e}$
[27]	2	4	0
[28]	0	6	0
Proposed SenseCrypt	2	4	0
**UnSigncrypt**			
**Schemes**	$T_{p}$	$T_{m}$	$T_{e}$
[27]	3	3	0
[28]	4	2	0
Proposed SenseCrypt	1	2	0

**Table 7:** Analysis of computational and communication overhead.

Reference	Computational Cost	Computational Overhead
[27]	$5T_{p}+6T_{m}$	$\left(n+1\right)\left|G_{1}\right|+n\left|m\right|$
[28]	$4T_{p}+8T_{m}$	$\left(n+1\right)\left|G_{1}\right|+n\left|m\right|$
Proposed SenseCrypt	$3T_{p}+6T_{m}$	$\left(n+1\right)\left|G_{1}\right|+n\left|m\right|$

**Table 8:** Time of Cryptographic Operations in SenseCrypt Framework.

Operations	Running Time	Descriptions
$T_{p}$	2.02 ms	The time for one pairing operation
$T_{m}$	0.1 ms	The time for a scalar point multiplication operation
